# Dopamine D3 Receptor Modulates Akt/mTOR and ERK_1/2_ Pathways Differently during the Reinstatement of Cocaine-Seeking Behavior Induced by Psychological versus Physiological Stress

**DOI:** 10.3390/ijms241311214

**Published:** 2023-07-07

**Authors:** Aurelio Franco-García, Rocío Guerrero-Bautista, Juana María Hidalgo, Victoria Gómez-Murcia, María Victoria Milanés, Cristina Núñez

**Affiliations:** 1 Group of Cellular and Molecular Pharmacology, Department of Pharmacology, CEIR Campus Mare Nostrum, University of Murcia, 30120 Murcia, Spain; aurelio.franco@um.es (A.F.-G.); rocio.guerrero@carm.es (R.G.-B.); jmhc@um.es (J.M.H.); milanes@um.es (M.V.M.); 2 Instituto Murciano de Investigación Biosanitaria (IMIB) Pascual Parrilla, 30120 Murcia, Spain

**Keywords:** cocaine-induced conditioned place preference, dopaminergic receptors, drug-seeking behavior, restraint, tail pinch, MEK_1/2_

## Abstract

Stress triggers relapses in cocaine use that engage the activity of memory-related nuclei, such as the basolateral amygdala (BLA) and dentate gyrus (DG). Preclinical research suggests that D3 receptor (D3R) antagonists may be a promising means to attenuate cocaine reward and relapse. As D3R regulates the activity of the Akt/mTOR and MEK/ERK_1/2_ pathways, we assessed the effects of SB-277011-A, a D3R antagonist, on the activity of these kinases during the reinstatement of cocaine-induced conditioned place preference (CPP) induced by psychological (restraint) and physiological (tail pinch) stress. Both stimuli reactivated an extinguished cocaine-CPP, but only restrained animals decreased their locomotor activity during reinstatement. Cocaine-seeking behavior reactivation was correlated with decreased p-Akt, p-mTOR, and p-ERK_1/2_ activation in both nuclei of restrained animals. While a D3R blockade prevented stress-induced CPP reinstatement and plasma corticosterone enhancement, SB-277011-A distinctly modulated Akt, mTOR, and ERK_1/2_ activation depending on the stressor and the dose used. Our data support the involvement of corticosterone in the SB-277011-A effects in restrained animals. Additionally, the ratios p-mTOR/mTOR and/or p-ERK_1/2_ /ERK_1/2_ in the BLA during stress-induced relapse seem to be related to the locomotor activity of animals receiving 48 mg/kg of the antagonist. Hence, our study indicates the D3R antagonist’s efficacy to prevent stress-induced relapses in drug use through distinct modulation of Akt/mTOR and MEK/ERK_1/2_ pathways in memory-processing nuclei.

## 1. Introduction

The cocaine market has undergone significant growth in the last 5 years. It has been estimated that 0.4% of the global population used cocaine in 2020. Hence, its consumption is a growing social problem due to the global prevalence of old and new users [1]. Substance-use disorders (SUDs) range from mild to severe based on the symptom criteria met by substance users, a characteristic of the most severe cases being the inability to maintain prolonged periods of abstinence, thus eventuating in frequent relapses [2].

Environmental stimuli play a significant role in the transition from mild to severe SUDs due to the occurrence of associative learning processes [3]. Thus, memory formation and retrieval are essential in the development of this condition. Currently, therapeutic strategies are focused on relapse prevention, although clinical trials on multiple potential drugs to address this issue have been unsuccessful [4]. Extinction therapy tries to deal with this matter by creating new memories that inhibit drug-paired associations developed during the drug-use period [5,6]. Nonetheless, this therapeutic approach has shown limited results since drug-related memories are not erased and can be reactivated by several triggers, such as stress, thus reinstating the previously extinguished drug-seeking behaviors [7,8,9].

Whereas environmental cues and drug priming seem to provoke relapse by evoking cocaine-paired memories [10], different stressful episodes might not follow the same neural and biochemical networks that lead to similar behavioral outcomes [11]. The restraint and tail-pinch paradigms are robust models to induce physiological and psychological stress, respectively [7,12]. Both of them have been extensively used to enhance the activation of the hypothalamic-pituitary-adrenal (HPA) axis and study drug-seeking related behaviors, as it has been previously reviewed [8].

Chronic exposure to some drugs of abuse is known to induce long-lasting alterations in the circuits critical for normal learning and memory processes. In the brain, the ventral tegmental area (VTA) sends dopaminergic projections, among other limbic areas, to the basolateral amygdala (BLA), that is linked to emotional memory processes, and to the hippocampus, in which the dentate gyrus (DG) plays a crucial role in episodic and spatial associations related to rewarding and aversive events in the environment [13,14,15,16,17,18]. Therefore, alterations in dopaminergic activity in the DG and BLA seem to work as modulators of drug-related memories that, in turn, are partially responsible for drug-seeking behaviors [9]. Among the dopaminergic receptors, the dopamine 3 receptor (D3R) appears to be particularly involved in reward, motivation, emotion, and learning, given its restricted distribution in the limbic system [19,20]. Accordingly, D3R has been proposed to play a large role in addiction-related behavior and, therefore, to be a potential target for relapse prevention in clinical therapy [21,22]. Several studies have shown the importance of D3R in relapse caused by stress, drug priming, and drug cues [23,24], while other findings provide conflicting results on the role of this receptor when its expression is suppressed [25,26]. In addition, D3R blockade diminishes the availability of these receptors and, consequently, D3R-coupled biochemical pathways might be altered [10,27,28]. D3R regulates the mitogen-activated protein-kinase (MEK)-signal-regulated kinases (ERK), phosphatidyl inositol 3-kinase (PI3K)-protein kinase B (Akt), and the mechanistic target of rapamycin (mTOR) intracellular signaling [28]. We have previously reported a beneficial effect of the antagonism of D3R to block drug-seeking reinstatement after social stress through modulation of the Akt/mTOR signaling pathway [10,27] but the impact of this antagonist on relapses in cocaine-seeking behavior caused by other kinds of stressful stimuli is yet to be determined. The mTOR pathway has been shown to play a major role in memory formation and recall through protein synthesis [29]. Since the downregulation of this cascade has been linked to hippocampus neurite outgrowth [30] and enhancement of cognitive tasks [31], its precise method of control remains unclear. Our laboratory has previously reported an upregulation of the mTOR pathway in the BLA and DG after D3R blockade and consequent prevention of psychosocial-stress-induced relapse in cocaine-seeking behavior [10]. Similarly, cumulative evidence has indicated the importance of the MEK/ERK_1/2_ pathway in long-term potentiation (LTP) and protein synthesis—critical events for memory formation and retrieval [32]. Hence, the aim of this work was to: (i) assess the impact of D3R blockade on the reinstatement of cocaine-seeking behavior induced by psychological and physiological stress after the extinction of a previously acquired conditioned place preference (CPP); and (ii) elucidate the activity of Akt/mTOR and MEK/ERK_1/2_ pathways in the BLA and DG and their possible relationship with the activity of the HPA axis during these events.

## 2. Results

### 2.1. D3R Blockade Impeded the Reactivation of Cocaine-Induced CPP Provoked by Both Restraint and Tail Pinch

**Figure 1:**
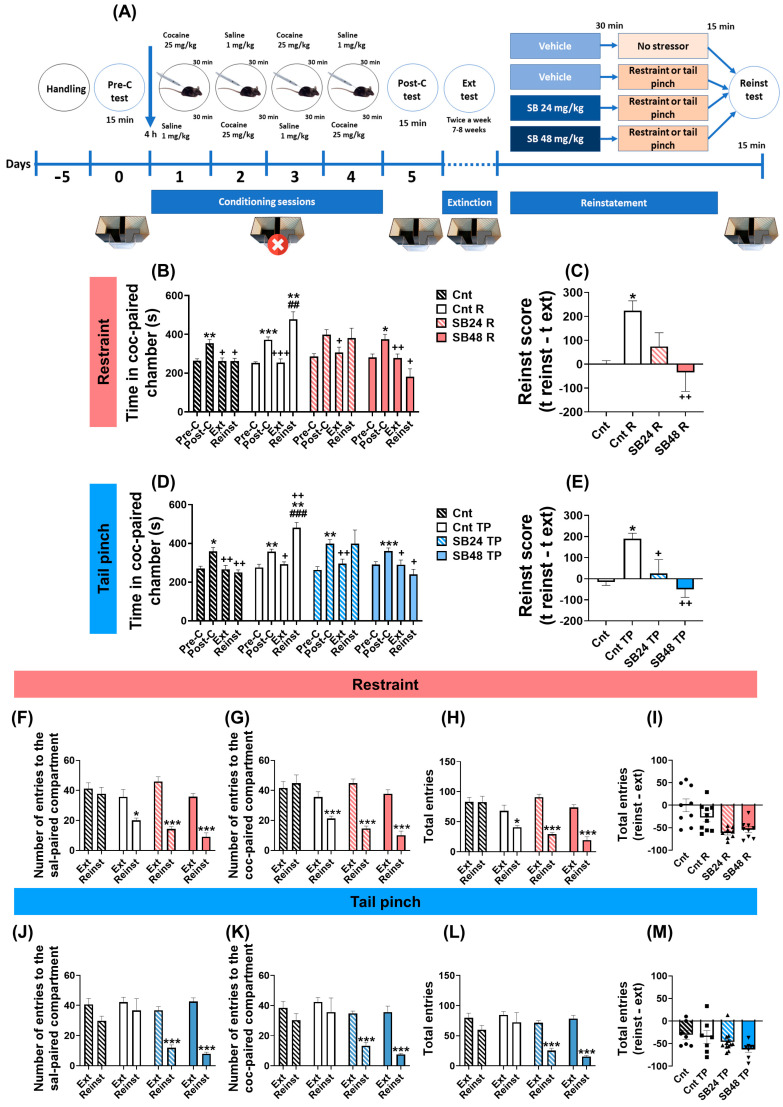
The antagonism of D3R blocked the reactivation of cocaine-induced CPP induced by both restraint and tail pinch. (**A**) Schematic of the experimental timeline and behavioral procedures. (**B**) Mean preference time spent in cocaine-paired chamber during pre-conditioning, post-conditioning, Extinction and Reinstatement of control mice (vehicle, no stressor; Cnt), animals that experienced restraint but did not receive the D3R antagonist (vehicle, restraint; Cnt R), and animals that experienced restraint and received the D3R antagonist at two different doses, 24 or 48 mg/kg, restraint; (SB24 R or SB48 R). Repeated measures one-way ANOVA showed differences among tests within Cnt group (F (2.182, 17.46) = 12.86; P = 0.0003), Cnt R group (F (1.461, 13.15) = 23.59; P = 0.0001), SB48 R (F (1.435, 12.91) = 7.776; P = 0.0098) but not SB24 R group (F (1.432, 10.02) = 3.418; P = 0.0845). * P < 0.05, ** P < 0.01, *** P < 0.001 vs. pre-conditioning; ^+^ P < 0.05, ^++^ P < 0.01, ^+++^ P < 0.001 vs. Post-conditioning; ^##^ P < 0.01 vs. Extinction (Tukey’s test). (**C**) Reinstatement score expressed as time in cocaine-paired chamber during Reinstatement test minus the same in Extinction test in Cnt, Cnt R, SB24 R and SB48 R animals. One-way ANOVA revealed significant differences among group means (F (3, 32) = 4.913; P = 0.0064). * P < 0.05 vs. Cnt; ^++^ P < 0.01 vs. Cnt R (Tukey’s test). (**D**) Mean preference time spent in cocaine-paired chamber during pre-conditioning, post-conditioning, Extinction, and Reinstatement of control mice (vehicle, no stressor; Cnt), animals that experienced tail pinch but did not receive the D3R antagonist (vehicle, tail pinch; Cnt TP), and animals that experienced tail pinch and received the D3R antagonist at two different doses SB-277011-B, 24 or 48 mg/kg, restraint; SB24 TP or SB48 TP). Repeated measures one-way ANOVA showed differences among tests within Cnt group (F (2.440, 17.08) = 14.24; P = 0.0001), Cnt TP group (F (1.917, 15.34) = 30.18; P < 0.0001), SB48 TP group (F (1.644, 11.51) = 6.606; P = 0.0154) but not SB24 TP group (F (1.314, 14.45) = 3.561; P = 0.0706). * P < 0.05, ** P < 0.01, *** P < 0.001 vs. pre-conditioning; ^+^ P < 0.05, ^++^ P < 0.01 vs. post-conditioning; ^###^ P < 0.001 vs. Extinction (Tukey’s test). (**E**) Reinstatement score expressed as time in cocaine-paired chamber during Reinstatement test minus the same time in Extinction test in Cnt, Cnt TP, SB24 TP and SB48 TP animals. One-way ANOVA revealed significant differences among group means (F (3, 30) = 5.505; P = 0.0039). * P < 0.05 vs. Cnt; ^+^ P < 0.05, ^++^ P < 0.01 vs. Cnt TP (Tukey’s test). (**F**–**H**) Locomotor activity during Extinction and Reinstatement tests for Cnt, Cnt R, SB24 R and SB48 R animals. Graphics show the number of entries to saline-paired (paired Student’s *t* test: Cnt (t_8_ = 0.5483, P = 0.5985), Cnt R (t_9_ = 2.930, P = 0.0167), SB24 R (t_7_ = 10.74, P < 0.0001) and SB48 R (t_8_ = 8.321, P < 0.0001)) (**F**) and cocaine-paired (paired Student’s *t* test: Cnt (t_8_ = 0.4024, P = 0.6979), Cnt R (t_8_ = 3.608, P = 0.0069), SB24 R (t_7_ = 11.87, P < 0.0001) and SB48 R (t_8_ = 8.168, P < 0.0001)) (**G**) compartments and total entries (paired Student’s *t* test: Cnt (t_8_ = 0.02346, P = 0.9819), Cnt R (t_9_ = 2.878, P = 0.0182), SB24 R (t_7_ = 14.27, P < 0.0001) and SB48 R (t_8_ = 8.602, P < 0.0001)) (**H**). (**F**–**H**) * P < 0.05, *** P < 0.001 vs. Extinction test (Tukey’s test). (**I**) Total entries score in Cnt, Cnt R, SB24 R and SB48 R animals, expressed as the difference in total entries during Reinstatement test minus total entries during Extinction test. One-way ANOVA: (F (3, 32) = 8.240; P = 0.0003). (**J**–**L**) Locomotor activity during Extinction and Reinstatement tests for Cnt, Cnt TP, SB24 TP and SB48 TP animals. Graphics show the number of entries to saline (sal)-paired (paired Student’s *t* test: Cnt (t_7_ = 1.619, P = 0.1495), Cnt TP (t_8_ = 0.5979, P = 0.5664), SB24 TP (t_11_ = 7.794, P < 0.0001) and SB48 TP (t_7_ = 14.24, P < 0.001) (**J**), cocaine (coc)-paired (paired Student’s *t* test: Cnt (t_7_ = 1.015, P = 0.3438), Cnt TP (t_8_ = 0.5979, P = 0.5377), SB24 TP (t_11_ = 6.390, P < 0.0001) and SB48 TP (t7 = 6.312, P = 0.0004) (**K**) compartments and total entries (paired Student’s *t* test: Cnt (t_7_ = 1.405, P = 0.2028), Cnt TP (t_8_ = 0.6641, P = 0.5253), SB24 TP (t_11_ = 7.386, P < 0.0001) and SB48 TP (t_7_ = 10.19, P < 0.0001) (**L**). (**J**–**L**) *** P < 0.001 vs. Extinction test. (**M**) Total entries score in Cnt, Cnt TP, SB24 TP and SB48 TP animals, expressed as the difference in total entries during Reinstatement test minus total entries during Extinction test. One-way ANOVA: (F (3, 30) = 2.297; P = 0.0977). Data are shown as mean ± S.E.M (*n* = 7–12 per group).

It is widely known that cocaine conditioning provokes a strong CPP in rodents [33]. Accordingly, all the mice used in this work spent significantly more time in the cocaine-associated compartment during the post-conditioning test versus the pre-conditioning test and, after the extinction sessions, the period that animals stayed in the drug-paired chamber in the extinction test was statistically similar to that in the pre-conditioning test (Figure 1B,D). We used two types of stressors, one psychological—restraint—and one physiological—tail pinch—to induce the reactivation of cocaine-seeking behavior. In contrast to the control mice, the animals subjected to an acute session of restraint or tail pinch spent significantly more time in the drug-paired chamber during the reinstatement test when compared with that in the extinction and pre-conditioning tests (Figure 1B,D). Additionally, as we had previously reported [34], the antagonism of D3R with 48 mg/kg but not 24 mg/kg of SB-277011-A, prevented the significant enhancement of the time spent by mice in the cocaine-associated compartment during the reinstatement test induced by any of these two stressors (Figure 1B,D). When we calculated the reinstatement score (as the difference of time that mice spent in the cocaine-paired compartment during the reinstatement test minus that in the extinction test), we found a significant increase in stressed animals in comparison with controls, and only the higher dose of the D3R antagonist was able to significantly block this effect (Figure 1C,E).

### 2.2. Restrained Animals but Not Those Subjected to Tail Pinch Reduced Their Locomotor Activity during the Reinstatement Test

Furthermore, we intended to study the locomotor activity of mice during the reactivation of cocaine-CPP induced by acute psychological or physiological stress. To accomplish that, we analyzed the number of entries to the cocaine- and saline-paired chambers and the sum of all of them during the reinstatement and extinction tests. Whereas there were no changes in the number of total entries as well as those to any of the compartments in control animals during both reinstatement and extinction tests, mice that were restrained significantly diminished their entries to any of the compartments of the CPP apparatus as well as the total entries during the reinstatement test when compared with the extinction test (Figure 1F–H). Nonetheless, this effect was not observed in mice that reinstated the cocaine-CPP after an episode of tail pinching (Figure 1I–K). In addition, after the administration of both doses—24 and 48 mg/kg—of SB-277011-B we observed a significant decrease in the activity of mice subjected to any of the stressors during the reinstatement test versus the extinction test (Figure 1F–H,J–L). To investigate whether D3R antagonism influenced the altered locomotor activity during the reactivation of cocaine-seeking behavior induced by restraint or tail pinch, we analyzed the difference between the number of total entries in the reinstatement test and that in the extinction test for each experimental group. However, there were no significant changes between animals pre-treated with vehicle before any of the stressful episodes and the mice injected with the D3R antagonist prior being subjected to restraint or the tail pinch (Figure 1I,M).

### 2.3. Corticosterone Plasma Concentration Is Related with the Reinstatement of Cocaine-CPP after Restraint but Not Tail Pinch Stress

**Figure 2:**
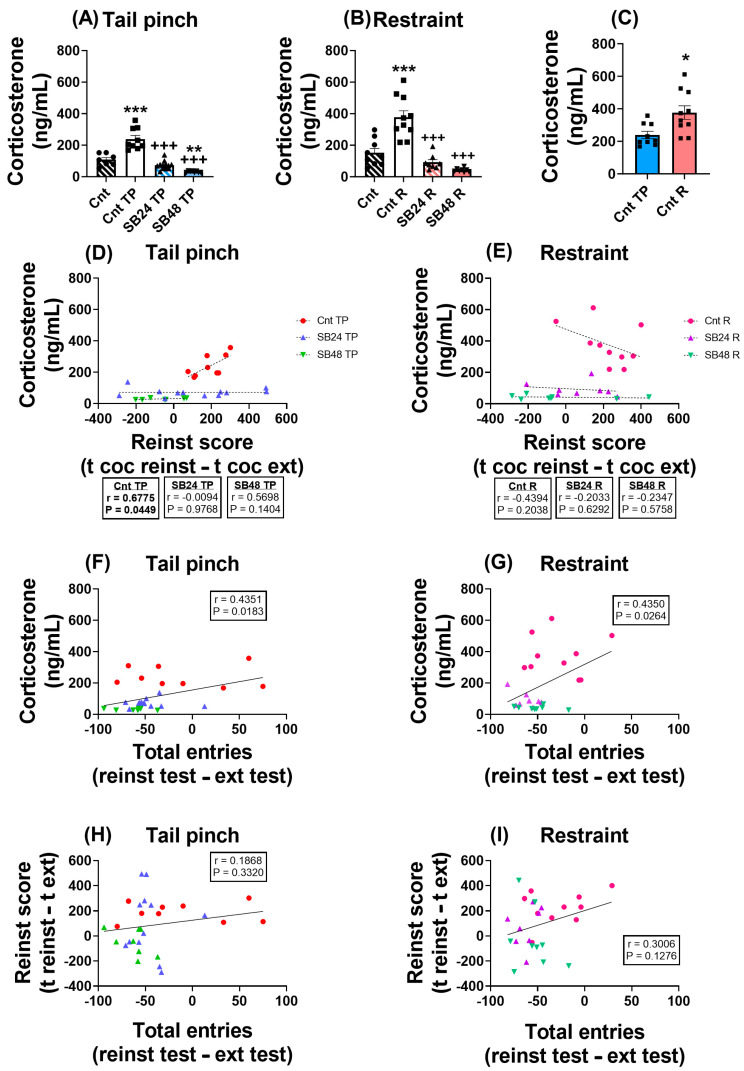
(**A**) Analysis of corticosterone levels in plasma of control mice (vehicle, no stressor; Cnt), animals that experienced tail pinch but did not receive the D3R antagonist (vehicle, tail pinch; Cnt TP), and animals that experienced tail pinch and received the D3R antagonist at two different doses (SB-277011-B, 24 or 48 mg/kg, tail pinch; SB24 TP or SB48 TP). One-way ANOVA: F (3, 32) = 42.59); P < 0.001. ** P < 0.01, *** P < 0.001 vs. Cnt, ^+++^ P < 0.001 vs. Cnt TP (Tukey’s test). Cohen’s *d* values: Cnt TP vs. Cnt [*d*: 2.38; effect size r: 0.77]; SB24 TP vs. Cnt TP [*d*: −3.21; effect size r: −0.85]; SB48 TP vs. Cnt TP [*d*: −4.30; effect size r: −0.91]; SB48 TP vs. Cnt [*d*: −2.80; effect size r: −0.81]. (**B**) Analysis of corticosterone levels in plasma of control mice (vehicle, no stressor; Cnt), animals that experienced restraint but did not receive the D3R antagonist (vehicle, restraint; Cnt R), and animals that experienced restraint and received the D3R antagonist at two different doses SB-277011-B,24 or 48 mg/kg, restraint; SB24 R or SB48 R). One-way ANOVA: F (3, 31) = 27.29; P < 0.001. *** P < 0.001 vs. Cnt, ^+++^ P < 0.001 vs. Cnt R (Tukey’s test). Cohen’s *d* values: Cnt R vs. Cnt [*d*: 2.04; effect size r: 0.71]; SB24 TP vs. Cnt TP [*d*: −2.88; effect size r: −0.82]; SB48 TP vs. Cnt TP [*d*: −3.58; effect size r: −0.87]. (**C**) Comparison of corticosterone levels in plasma between Cnt TP and Cnt R groups. Unpaired Student’s *t* test: t_17_ = 2.809, P = 0.0121. * P < 0.05 vs. Cnt TP. Cohen’s *d* values: Cnt R vs. Cnt TP [*d*: 1.31; effect size r: 0.55]. (**D**) Correlation analysis between plasmatic corticosterone and reinstatement score in tail pinched animals, measured as the time spent in cocaine-paired chamber during reinstatement test minus the same during extinction test. (**E**) Correlation analysis between plasmatic corticosterone and reinstatement score in restrained animals. (**F**) Correlation analysis between plasmatic corticosterone and total entries in tail pinched animals, measured as the total entries during reinstatement test minus those during extinction test. (**G**) Correlation analysis between plasmatic corticosterone and total entries in restrained animals. (**H**) Correlation analysis between reinstatement score and total entries in tail pinched animals. (**I**) Correlation analysis between reinstatement score and total entries in restrained animals. Correlation analyses were revealed through Pearson’s test. Data are shown as mean ± S.E.M (*n* = 7–12 per group).

To investigate whether the hormonal response to stress affects the reinstatement of cocaine-CPP, we quantified corticosterone plasma concentration after the reinstatement test in all the experimental groups. Similar to our previous work [34], we found that after the reactivation of cocaine-CPP induced by both restraint or the tail pinch, the levels of corticosterone in plasma significantly increased when compared with control animals, and that the administration of both 24 and 48 mg/kg of the D3R antagonist blocked this enhancement (Figure 2A,B). Curiously, we detected that the corticosterone concentration of restrained mice was significantly higher in comparison with animals that were subjected to the tail pinch to induce the reactivation of cocaine-CPP (Figure 2C). Moreover, corticosterone plasma concentration was highly positively correlated with the reinstatement score of animals that received vehicle and were subjected to the tail pinch to induce the reactivation of cocaine-seeking behavior, whilst this correlation was not significant in mice that were injected with the vehicle and restrained (Figure 2D,E). In addition, there were not significant correlations between the reinstatement score of the animals injected with both doses of SB-277011-A prior the tail pinch or restraint episode and their corticosterone plasma levels (Figure 2D,E), thus suggesting that D3R would modulate this association at least when the relapse in cocaine-CPP is triggered by a physiological stressor. Moreover, corticosterone plasma levels moderately correlated positively with the difference of total entries between the reinstatement and extinction tests of mice receiving D3R antagonist or its vehicle before being subjected to a psychological or physiological stressor to induce the relapse in cocaine-CPP (Figure 2F,G). However, this difference in entries did not correlate with the reinstatement score of mice restrained or tail pinched after being administered with vehicle or SB-277011-A (Figure 2H,I).

Collectively, these data appear to indicate that, although stress could augment the locomotor activity of animals, this activity is unlikely to be decisive for the reinstatement of cocaine-seeking behaviors.

### 2.4. Restraint but Not Tail Pinch Decreased the Phosphorylation and/or Activation of the Akt/mTOR and ERK_1/2_ Pathways in the BLA of Mice That Relapsed in Cocaine-CPP

We next studied the activation, as the ratio between the phosphorylated (p) and total protein, of ERK_1/2_, Akt, and mTOR since they have long been known to be related to the plastic changes that occur during the formation and retrieval of different kind of memories [29,32], and are regulated by D3R [10,27,28] in the BLA and the DG.

**Figure 3:**
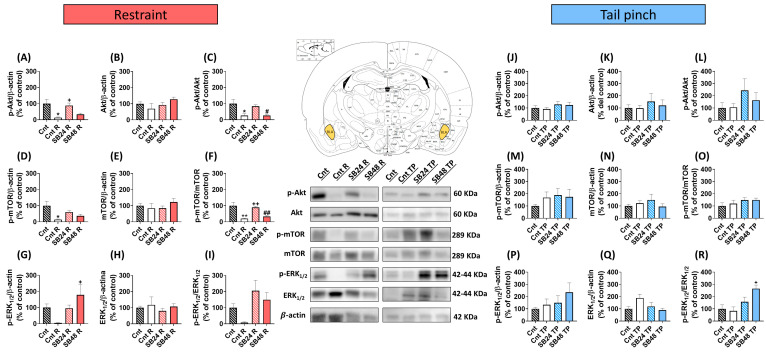
Basolateral amygdala analysis in restraint and tail-pinch-stress paradigms. (**A**–**C**) Semiquantitative analysis and representative immunoblot of p-Akt/β-actin, Akt/β-actin, and p-Akt/Akt levels in restraint paradigm. One-way ANOVA: F (3, 21) = 5.454; P = 0.0062 (**A**); F (3, 21) = 1.774; P = 0.1829 (**B**); F (3, 21) = 6.916; P = 0.002 (**C**). (**D**–**F**) Semiquantitative analysis and representative immunoblot of p-mTOR/β-actin, mTOR/β-actin, and p-mTOR/mTOR levels in restraint paradigm. One-way ANOVA: F (3, 21) = 4.588 (**D**); F (3, 22) = 0.9630, P = 0.4278 (**E**); F (3, 22) = 11.79; P < 0.0001 (**F**). (**G**–**I**) Semiquantitative analysis and representative immunoblot of p-ERK_1/2_/β-actin, ERK_1/2_/β-actin, and p-ERK_1/2_/ERK_1/2_ levels in restraint paradigm. One-way ANOVA: F (3, 19) = 2.94; P = 0.0494 (**G**); F (3, 21) = 0.5587; P = 0.6481 (**H**), F (3, 22) = 2290; P = 0.1064 (**I**). (**A**–**I**) * P < 0.05, ** P < 0.01 vs. Cnt; ^+^ P < 0.05, ^++^ P < 0.01 vs. Cnt R; ^#^ P < 0.05, ^##^ P < 0.05 vs. SB24 R (Tukey’s test). (**J**–**L**) Semiquantitative analysis and representative immunoblot of p-Akt/β-actin, Akt/β-actin and p-Akt/Akt levels in tail-pinch paradigm. One-way ANOVA: F (3, 23) = 0.7468; P = 0.5353 (**J**); F (3, 23) = 0.3078; P = 0.8195 (**K**); F (3, 24) = 0.9801; P = 0.4186 (**L**). (**M**–**O**) Semiquantitative analysis and representative immunoblot of p-mTOR/β-actin, mTOR/β-actin, and p-mTOR/mTOR levels in tail-pinch paradigm. One-way ANOVA: F (3, 24) = 0.4483; P = 0.7208 (**M**); F (3, 24) = 0.6504; P = 0.5904 (**N**); F (3, 24) = 1.067; P = 0.3817 (**O**). (**P**–**R**) Semiquantitative analysis and representative immunoblot of p-ERK_1/2_/β-actin, ERK_1/2_/β-actin and p-ERK_1/2_/ERK_1/2_ levels in tail-pinch paradigm. One-way ANOVA: F (3, 22) = 0.8227; P = 0.4953 (**P**); F (3, 23) = 2.744; P = 0.0663 (**Q**); F (3, 23) = 6.016; P = 0.0035 (**R**). (**J**–**R**) ^+^ P < 0.05 vs. Cnt TP (Tukey’s test). Data are shown as mean ± S.E.M (*n* = 4–8 per group).

**Figure 4:**
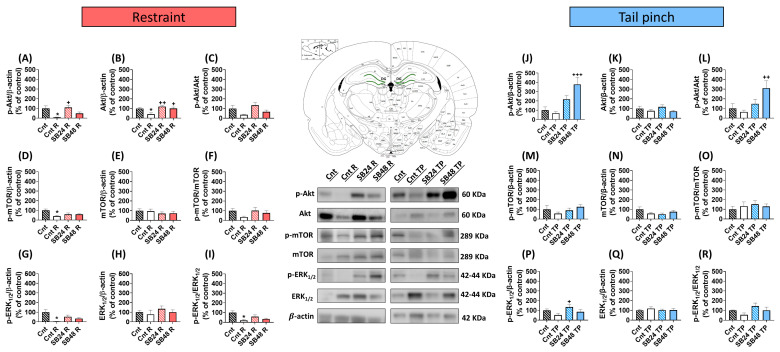
Dentate gyrus analysis in restraint and tail-pinch-stress paradigms (**A**–**C**) Semiquantitative analysis and representative immunoblot of p-Akt/β-actin, Akt/β-actin, and p-Akt/Akt levels in restraint paradigm. One-way ANOVA: F (3, 24) = 3838; P = 0.0224 (**A**); F (3, 24) = 4.951; P = 0.0081 (**B**); F (3, 24) = 2.086; P = 0.1287 (**C**). (**D**–**F**) Semiquantitative analysis and representative immunoblot of p-mTOR/β-actin, mTOR/β-actin, and p-mTOR/mTOR levels in restraint paradigm. One-way ANOVA: F (3, 24) = 3.663; P = 0.0264 (**D**); F (3, 24) = 0.5961; P = 0.6237 (**E**); F (3, 23) = 0.9933; P = 0.4135 (**F**). (**G**–**I**) Semiquantitative analysis and representative immunoblot of p-ERK_1/2_/β-actin, ERK_1/2_/β-actin and p-ERK_1/2_/ERK_1/2_ levels in restraint paradigm. One-way ANOVA: F (3, 23) = 4.390; P = 0.0139 (**G**); F (3, 24) = 0.7024; P = 0.5599 (**H**); F (3, 23) = 5.051; P = 0.0078 (**I**). (**A**–**I**) * P < 0.05 vs. Cnt; ^+^ P < 0.05, ^++^ P < 0.01 vs. Cnt R (Tukey’s test). (**J**–**L**) Semiquantitative analysis and representative immunoblot of p-Akt/β-actin, Akt/β-actin, and p-Akt/Akt levels in tail-pinch paradigm. One-way ANOVA: F (3, 23) = 9.915; P = 0.0002 (**J**); F (3, 23) = 1.442; P = 0.2563 (**K**); F (3, 24) = 4.678; P = 0.0104 (**L**). (**M**–**O**) Semiquantitative analysis and representative immunoblot of p-mTOR/β-actin, mTOR/β-actin, and p-mTOR/mTOR levels in tail-pinch paradigm. One-way ANOVA: F (3, 24) = 1.513; P = 0.2366 (**M**); F (3, 24) = 1.806; P = 0.1730 (**N**); F (3, 22) = 0.2821; P = 0.8377 (**O**). (**P**–**R**) Semiquantitative analysis and representative immunoblot of p-ERK_1/2_/β-actin, ERK_1/2_/β-actin, and p-ERK_1/2_/ERK_1/2_ levels in tail-pinch paradigm. One-way ANOVA: F (3, 24) = 2.817; P = 0.0606 (**P**); F (3, 24) = 0.3584; P = 0.7835 (**Q**); F (3, 24) = 2.188; P = 0.1157 (**R**). (**J**–**R**) ^+^ P < 0.05, ^++^ P < 0.01, ^+++^ P < 0.001 vs. Cnt TP (Tukey’s test). Data are shown as mean ± S.E.M (*n* = 4–8 animals per group).

In the BLA of mice that were restrained in order to reactivate the cocaine-induced CPP we detected significant decreases versus controls of the levels of p-Akt and p-mTOR, but not of p-ERK_1/2_, (Figure 3A,D,G), that paralleled with invariable amounts of total Akt, mTOR and ERK_1/2_ (Figure 3B,E,H). Thus, the p-Akt/Akt and p-mTOR/mTOR ratios were statistically diminished in this area (Figure 3C,F) as opposed to p-ERK_1/2_/ERK_1/2_ (Figure 3I). On the contrary, in the BLA of mice subjected to the tail pinch before the reinstatement test, we did not observed alterations in the levels of p-Akt, p-mTOR or p-ERK_1/2_ (Figure 3J,M,P) nor in their total protein levels (Figure 3K,N,Q). Hence, the ratio of these kinases during psychological- and physiological-stress-induced relapse in cocaine-CPP did not differ from the control group (Figure 3L,O,R). Similarly, in the DG of restrained animals prior the reinstatement test we found significant decreases in the levels of p-Akt, p-mTOR and p-ERK_1/2_ in comparison with the control animals (Figure 4A,D,G). In addition, we observed lower levels of total Akt, but not of total mTOR nor ERK_1/2_ (Figure 4B,E,H). As a result, p-ERK_1/2_/ERK_1/2_, but not p-Akt/Akt nor p-mTOR/mTOR, significantly decreased in the DG after the reinstatement of cocaine-CPP induced by restraint in comparison with the control group (Figure 4C,F,I). On the contrary, animals subjected to an acute session of tail pinching to induce the relapse in cocaine-CPP did not exhibit alterations in p-Akt, p-mTOR nor p-ERK_1/2_ levels in the DG versus controls (Figure 4J,M,P). Additionally, no differences in total Akt, mTOR, and ERK_1/2_ between Cnt and Cnt-TP groups were detected (Figure 4K,N,O). As a consequence, p-Akt/Akt, p-mTOR/mTOR, and p-ERK_1/2_/ERK_1/2_ ratios did not change significantly in this hippocampal region after the reinstatement of cocaine-CPP induced by a physiological stressor when compared with controls (Figure 4L,O,R).

### 2.5. Distinct Doses of D3R Antagonist Modulate Differently the Activity of the Akt/mTOR and ERK_1/2_ Pathways in the BLA and DG during the Reactivation of Cocaine-CPP

The blockade of D3R had different effect on the activity of Akt, mTOR, and ERK_1/2_ during the reinstatement of cocaine-CPP depending on the triggering stressor and the dose of antagonist administered.

The BLA from animals that received 24 mg/kg of SB-277011-A before being restrained to induce the relapse in cocaine-seeking behavior exhibited significantly higher levels of p-Akt compared to restrained mice that were injected with vehicle (Figure 3A). In contrast, p-mTOR and p-ERK_1/2_ levels in the BLA of mice that received 24 mg/kg of the D3R blocker before being restrained were not statistically different to those of mice injected with vehicle (Figure 3D,G). Total Akt, mTOR, and ERK_1/2_ levels were not affected by the blockade of D3R with the lower dose of SB-277011-A before the restraint episode (Figure 3B,E,F). When we calculated the phosphorylation ratio of these kinases, we detected that p-mTOR/mTOR but not p-Akt/Akt nor p-ERK_1/2_/ERK_1/2_ increased significantly in the BLA of mice that received the lower dose of the D3R antagonist prior restraint versus restrained animals injected with vehicle.

Conversely, the dose of 24 mg/kg of SB-211077-A during the reinstatement of cocaine-CPP induced by the tail pinch did not modify p-Akt, p-mTOR, and p-ERK_1/2_ levels (Figure 3J,M,P) nor total Akt, mTOR, and ERK_1/2_ levels (Figure 3K,N,Q) in the BLA when compared with Cnt-TP group and, consequently, we did not detect significant alterations of the ratios p-Akt/Akt, p-mTOR/mTOR, and p-ERK_1/2_/ERK_1/2_ in this area regarding animals that received vehicle instead the D3R blocker before the tail pinch session (Figure 3L,O,R).

The DG of animals that were administered with 24 mg/kg of SB-277011-A before being restrained to induce the reinstatement of the cocaine-CPP exhibited significantly higher levels of p-Akt, but not p-mTOR or p-ERK_1/2_ in comparison with mice that were injected with vehicle instead the D3R antagonist prior the stressful stimulus (Figure 4A,D,G). Whereas no alterations were found in mTOR and ERK_1/2_ levels, total Akt was significantly higher in the DG of animals injected with 24 mg/kg of the D3R antagonist in comparison with mice receiving vehicle prior the restraint episode (Figure 4B,E,H). As a result, we did not find significant changes in the ratios of p-Akt/Akt, p-mTOR/mTOR and p-ERK_1/2_/ERK_1/2_ in the DG of mice that received the lower dose of the D3R antagonist versus those injected with vehicle before being restrained (Figure 4C,F,I).

On the other hand, after the administration of the lower dose of the D3R antagonist before the tail pinching episode no significant differences in the levels of p-Akt and p-mTOR in comparison with mice injected with vehicle were detected (Figure 4J,M). In contrast, p-ERK_1/2_ levels were significantly augmented (Figure 4P). Total proteins levels in this region were not affected by the administration of 24 mg/kg of SB-277011-B prior to the tail pinch (Figure 4K,N,Q), and the resultant phosphorylation ratio of these kinases in the DG of mice that were injected with 24 mg/kg of SB-277011-B and subjected to acute physiological stress before the reinstatement test did not differ significantly of those of animals receiving vehicle instead of the D3R antagonist (Figure 4L,O,R).

In the BLA of mice that were injected with the higher dose of SB-277011-B before the restraint session p-Akt and p-mTOR levels were similar to those of vehicle-injected restrained animals (Figure 3A,D) in contrast with p-ERK_1/2_ levels, that were significantly higher than those of animals that received vehicle before the restraint episode (Figure 3G). Total Akt, mTOR, and ERK_1/2_ in the BLA under this experimental condition remained unchanged (Figure 3B,E,H), which resulted in a significant decrease of p-Akt/Akt and p-mTOR/mTOR in the BLA in comparison with animals injected with 24 mg/kg of SB-277011-B (Figure 3C,F), while p-ERK_1/2_/ERK_1/2_ were statistically similar in comparison with this group (Figure 3I).

The administration of the higher dose of the D3R blocker prior the tail pinch did not modify p-Akt, p-mTOR and p-ERK_1/2_ (Figure 3J,M,P) nor total Akt, mTOR and ERK_1/2_ levels (Figure 3K,N,Q) in the BLA. Therefore, the phosphorylation ratio of Akt and mTOR remained unaltered (Figure 3L,O) in this nucleus during the blockade of cocaine-CPP reactivation induced by a physiological stressor. Nevertheless, as p-ERK_1/2_ levels showed a tendency to increase, p-ERK_1/2_/ERK_1/2_ was significantly incrased in the BLA of animals that received 48 mg/kg of the D3R antagonist with regard to those receiving vehicle before the tail-pinch session (Figure 3R).

In the DG of animals injected with the higher dose of SB-277011-B before being restrained we did not detect significant differences in the levels of p-Akt, p-mTOR, and p-ERK_1/2_ when compared with those of animals that received vehicle or the lower dose of SB-277011-B prior the stressful stimulus (Figure 4A,D,G). In contrast to the total levels of Akt in the DG of mice injected with the higher dose of the D3R blocker prior restraint, that were significantly enhanced in comparison with those of animals injected with vehicle (Figure 4B), total mTOR and ERK_1/2_ remained unaltered (Figure 4E,H). As a result, we did not detect significant differences in the phosphorylation ratio of Akt, mTOR, and ERK_1/2_ in the DG after the administration of 48 mg/kg of the D3R antagonist in comparison with those of mice that received the lower dose of SB-277011-B or vehicle before being restrained (Figure 4C,F,I).

On the other hand, in the DG of mice injected with 48 mg/kg of SB-277011-B before an acute episode of physiological stress, we did not find significant changes in p-mTOR and p-ERK_1/2_, (Figure 4M,P) nor total ERK_1/2_ and mTOR levels (Figure 4N,Q) in comparison with tail-pinched animals receiving vehicle or 24 mg/kg of the D3R antagonist and, thus, the activation of ERK_1/2_ and mTOR in the DG of these animals remained unchanged (Figure 4O,R). In contrast, p-Akt levels significantly increased in the DG of animals injected with the higher dose of the D3R blocker and subjected to tail pinching when compared with those of tail-pinched animals that received the vehicle (Figure 4J). As total Akt did not change (Figure 4K), p-Akt/Akt statistically increased in the DG of mice that received 48 mg/kg of the D3R antagonist to prevent the reactivation of cocaine-CPP induced by the tail pinch (Figure 4L).

### 2.6. Corticosterone Plasma Concentration Is Related with the Activation of Akt/mTOR Pathway but Not ERK_1/2_ in the BLA and DG

**Figure 5:**
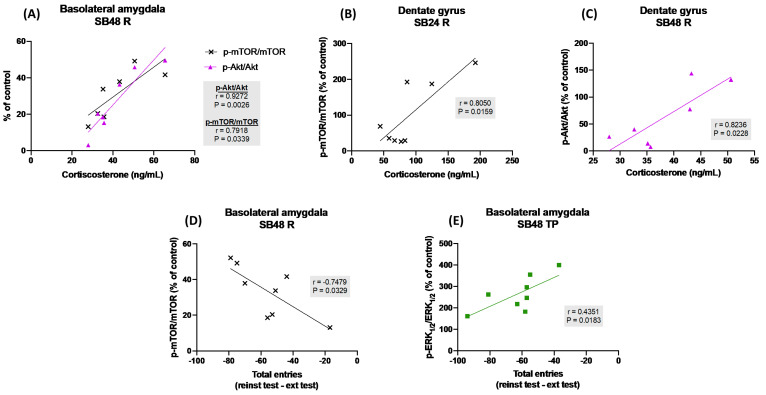
(**A**) Correlation analysis of corticosterone in plasma of animals that experienced restraint and received the D3R antagonist at 48 mg/kg (SB48 R); p-Akt/Akt and p-mTOR/mTOR levels in basolateral amygdala. (**B**) Correlation analysis of corticosterone in plasma of animals that experienced restraint and received the D3R antagonist at 24 mg/kg (SB24 R) and p-mTOR/mTOR levels in dentate gyrus. (**C**) Correlation analysis of corticosterone in plasma and p-Akt/Akt levels in dentate gyrus of SB48 R mice. (**D**) Correlation analysis of total entries, expressed as the total entries during reinstatement test minus those during extinction test, and p-mTOR/mTOR in basolateral amygdala of SB48 R animals. (**E**) Correlation analysis of total entries and p-ERK_1/2_/ERK_1/2_ levels in basolateral amygdala of animals that experienced tail pinch and received the SB-277011-A at 48 mg/kg (SB48 TP). Correlation analyses were performed through Pearson’s test (*n* = 4–8 animals per group).

We then studied, by means of Pearson’s correlation analysis, the possible relationship between the ratio of phosphorylated/total protein levels of Akt, mTOR and ERK_1/2_ in the BLA and the DG and the reinstatement of cocaine-seeking behaviors induced by psychological or physiological stress, but we did not find any significant correlations between those activities and the time spent by mice in the cocaine-paired compartment during the reins test nor the reinstatement score (data not showed). Next, we investigated whether the phosphorylation ratio of these kinases were related with the corticosterone plasma concentration of animals subjected to acute psychological or physiological stress to induce the reactivation of cocaine-seeking, and we detected high and positive correlations with p-Akt/Akt and p-mTOR/mTOR in the BLA of mice that were injected with 48 mg/kg of the D3R blocker and therefore did not relapse in cocaine-CPP after being restrained (Figure 5A), with p-mTOR/mTOR in the BLA of mice that received 24 mg/kg of the D3R antagonist prior the psychological stressor (Figure 5B) and with p-Akt/Akt in the DG of animals that were administered with 48 mg/kg of SB-277011-A before the same stimulus (Figure 5C). Finally, we analyzed whether Akt, mTOR and ERK_1/2_ phosphorylation ratios in the BLA and the DG were correlated with the locomotor activity (measured as the difference in the number of total entries to both saline- and cocaine-paired compartments during the reinstatement test minus that during the extinction test) of animals. We found that this difference of entries, on the one hand, significantly correlated highly and negatively with and p-mTOR/mTOR in the BLA of mice that received 48 mg/kg of the D3R antagonist before being restrained and, thus, did not reinstate the cocaine-CPP (Figure 5D) and, on the other hand, was significantly positively correlated with p-ERK_1/2_/ERK_1/2_ in the BLA of mice that were injected with the greater dose of SB-277011-B before the physiological stress episode and, consequently, did not reactivate cocaine-CPP (Figure 5E).

## 3. Discussion

In line with our previous investigations [34], this study has shown that an episode of restraint or tail pinching reactivated the previously extinguished cocaine-associated seeking behavior responsible for the CPP. In addition, this work reveals that the locomotor activity of animals restrained to induce the reinstatement of cocaine-CPP was diminished regarding the extinction stage in contrast to mice that were tail-pinched, that displayed similar locomotor activity during the extinction and reinstatement tests. Besides the significant differences in their blood corticosterone levels, this disparity might also be related with the significant decreases in p-Akt, p-mTOR, and p-ERK_1/2_ in the BLA and DG observed in animals that were restrained, that were not found when the relapse in cocaine-CPP was provoked by tail pinching. The present study also confirms our previous data [34] showing that the blockade of D3R prevented the reactivation of cocaine-seeking behavior in mice subjected to an episode of psychological or physiological stress as well as the parallel-enhanced corticosterone plasma concentration, and additionally shows that the effect of D3R antagonism on the phosphorylation of Akt, mTOR, and ERK_1/2_ in the BLA and DG differs between the two kinds of stressors used to reinstate the cocaine-CPP. While the blockade of D3R prior to the tail pinch increased the activation of some of these kinases in the amygdalar and hippocampal areas analyzed, the dose of antagonist administered seemed to influence the activation of the Akt/mTOR and MEK/ERK_1/2_ pathways when restraint triggered the relapse in cocaine-seeking behaviors. Corticosterone may be, in part, related with these variations as we found high correlations between its plasma levels and the phosphorylation ratio of mTOR and/or Akt in both the BLA and DG of animals that were injected with the D3R antagonist before being restrained. Moreover, our correlation analyses detected that the locomotor activity of animals injected with the higher dose of the D3R antagonist, SB-277011-A, might be related with mTOR phosphorylation ratio in the BLA when the reactivation of cocaine-CPP was induced by restraint, and with p-ERK_1/2_ phosphorylation ratio in the same brain region when the reinstatement of cocaine-CPP was provoked by an acute session of tail pinch.

Although stress is a recognized risk factor for relapses in drug use, not all the stressors were able to induce the reinstatement of drug-seeking behaviors in different animal models for SUD research [8]. The present study confirms tail pinch as an effective stressor to reinstate this behavior, as was first reported in our previous investigation [34]. In agreement with previous literature [12], our data have revealed that both psychological and physiological stressors activated the HPA axis, thus increasing glucocorticoid plasma levels. The higher corticosterone plasma concentrations of restrained animals versus those of tail-pinched mice might be due to the different anatomical substrates implicated in the psychological and physiological stress response [12]. Restraint sessions in retainers similar to the ones used in the present study with durations ranging from 1 h to 12 h have been considered previously as mild stress [35,36,37]. On the other hand, tail-pinch sessions ranging in duration from 5 to 15 min are described as mild stressors in rats and mice [38,39,40,41]. Nonetheless, the influence of a different magnitude of the stressful stimuli in the greater glucocorticoid blood levels of restrained animals cannot be ruled out. Moreover, unlike with restrained mice, the corticosterone blood concentrations of tail-pinched mice correlated highly with their reinstatement score. Altogether, these data might indicate a different degree of activation of the hypothalamic stress system depending upon the kind of stressor and might suggest that, once over certain glucocorticosteroids plasma levels, this parameter does not influence cocaine-seeking behaviors. New studies using different types of stressors or increased exposure to stress for inducing relapses in drug use would aid in answering this question. Nevertheless, corticosterone blood concentration does not seem to significantly affect locomotor activity during the relapse in cocaine-CPP induced by tail pinch versus restraint. Additionally, our data exhibited longer periods of restrained mice remaining in the cocaine-paired chamber during the reinstatement test than those during the extinction test, in contrast to mice that were physiologically stressed. This fact might point out an enhanced emotional value of the drug-associated context gained when the reinstatement of CPP is induced by psychological versus physiological stress.

In the drug-induced CPP paradigm, the rewarding effects of the addictive compound are associated with Pavlovian conditioning with a specific environment, and the reactivation of this preference is directly related to the recall of the pleasurable memories paired with the drug [9,42]. The retrieval of distinct kinds of memories has been associated with alterations in the activity of Akt/mTOR and MEK/ERK_1/2_ pathways in several brain areas [29,32], although the manner in which they change remains contentious. Traditionally, the activation of ERK_1/2_ and Akt/mTOR pathways, in their role in synaptoplasticity, has been considered vital for hippocampal declarative memory retrieval [43]. Nonetheless, recently, diminished mTOR and/or Akt phosphorylation after the recall of drug-associated aversive and rewarding memories in several limbic nuclei have been reported [10,27,44,45]. The different pattern of activation of the kinases studied in this work in the BLA and DG during the reactivation of cocaine-CPP after distinct kinds of triggering stressful stimuli could be explained by the different signaling molecules and neurocircuits that mediate their actions. While the physiological stressors act, at least partially, by means of defined receptor systems, the psychological and psychosocial stressful stimuli are processed through less specific exteroceptors and/or somatic inputs with emotional components [12,46]. In agreement, we report that a psychological stressor, but not a physiological one, altered the activation of Akt/mTOR and ERK_1/2_ pathways during the relapse in cocaine-CPP in the BLA, which is specifically involved in processing emotional memories. Moreover, the downregulation of Akt/mTOR and MEK/ERK_1/2_ pathways during the reactivation of the cocaine-seeking behavior induced by restraint was concordant with what was observed in these brain regions when the recall of drug-induced CPP was provoked by a psychosocial stressor [10]. On the other hand, the effects of the tail pinch have been shown to be mediated by dopaminergic and orexinergic neurotransmission systems and to influence cognitive processes [41,47,48,49]. Nevertheless, these effects have been reported to be integrated in the ventral hippocampus and medial amygdala [41,50], which might explain the lack of activation observed in the DG and BLA during the reinstatement of cocaine-CPP induced by this stressor. Additionally, as stated before, a distinct intensity of any of the stressors used to induce the reinstatement of cocaine-CPP cannot be dismissed.

Dopaminergic inputs from the VTA to its projection areas are vital for the reinstatement of drug use [51]. Furthermore, an increased extracellular dopamine concentration has been associated with acute restraint [52] and tail pinching [53]. In particular, D3R has been proposed to have a critical role in the maintenance of the behavioral outcomes of drug addiction [54]. Accordingly, it has been reported that D3R blockade prevented relapse in drug-seeking behaviors provoked by stress [10,23,24,27,34,55,56,57]. The BLA and DG receive direct dopaminergic innervation from the VTA [14,15,16]. In addition, we recently detected D3R expression in glutamatergic and GABAergic neurons in both areas [10]. However, neither those data nor the present results allow us to differentiate the role of presynaptic D3R autoreceptors from that of postsynaptic D3R heteroreceptors. On this matter, it has been proposed that D3R likely plays only a minor role as an autoreceptor and the vast majority of autofeedback inhibition is thought to be mediated through the D2R [58]. Considering D3R predominantly as a heteroreceptor and assuming its inhibitory character, D3R activation during relapse in cocaine use would lead to a decrease of the activity of the dopaminoceptive cells. Although confusing, these data would agree with reports indicating that the inhibition of BLA glutamatergic neurons is needed for the reinstatement of nicotine-induced CPP [59], as D3R could participate in this inhibition. Another possibility, given that we have also previously detected D3R in GABAergic interneurons of the BLA and DG [10], is that during the reinstatement of cocaine-CPP, D3R activation would lead to the inhibition of GABAergic cells and the subsequent activation of the neurons receiving GABAergic inputs in these areas. In our previous study, we showed that D3R blockade diminished the availability of these receptors during the reinstatement of cocaine-CPP induced by social stress [10]. Consequently, the activity of D3R-coupled biochemical pathways is expected to be affected after its antagonism [10,27,28]. SB-277011-A administration to untreated mice has been shown not to affect the activation of the Akt/mTOR and MEK_1/2_/ERK_1/2_ pathways [28,60]. Our data reflect different effects of D3R antagonism on Akt, mTOR, and ERK_1/2_ activation during the reinstatement of cocaine-CPP induced by two different stressors. Akt phosphorylation at Ser 473 results in its full activation and, consequently, to leads to the phosphorylation of its downstream substrates, mTORC1 being among them [61]. The unchanged levels of p-mTOR in both the BLA and DG despite Akt phosphorylation at Ser 473 in animals that received the D3R blocker before the tail pinch session might respond to the complex modulation of the Akt/mTOR signaling pathway, that is composed by more than 250 components and 478 links between them to participate in several cellular events [62]. The MEK/ERK_1/2_ signal has been speculated to cooperate with Akt in some cellular events and compensate the latter’s function when it is deactivated [63]. Hence, our results might indicate that D3R participates in the reactivation of cocaine-CPP provoked by physiological stress through different and/or balanced modulation of ERK_1/2_ and Akt activity. Additionally, different signaling pathways whose activity is under D3R regulation, such as adenylate cyclase (AC)-cAMP-protein kinase A (PKA), among others [51], would probably participate in this action. Additionally, and as stated before, a distinct intensity of the stressors used in this work to reinstate cocaine-CPP cannot be dismissed and might participate in these discrepancies.

In contrast, our data indicate a more intricate involvement of D3R in the reinstatement of cocaine-seeking behaviors induced by psychological stress given that the lower dose of SB-277011-A, that was ineffective to prevent the reinstatement of cocaine-CPP, produced a general increase of the phosphorylation of Akt, mTOR and ERK_1/2_ in both the BLA and DG, whereas the administration of 48 mg/kg of the D3R antagonist, that efficiently blocked the restraint-evoked reactivation of the cocaine-seeking behavior, affected distinctly to the phosphorylation of these kinases in both the amygdalar and hippocampal areas analyzed. These data are, to a certain extent, similar to what we observed in our previous investigations. The administration of doses of SB-277011-A that did not inhibit the reinstatement of cocaine-CPP induced by social stress increased p-mTOR levels in several dopaminoceptive nuclei in comparison with the stressed group treated with the vehicle. that relapsed in cocaine-seeking behavior, while higher doses of the D3R blocker, that efficiently inhibited the social-stress-induced reactivation of cocaine-CPP, was correlated with higher p-mTOR levels in these areas than those observed after the administration of the lower dose of the antagonist and were not different from those of the animals that relapsed in cocaine-seeking behavior [10,27]. It seems illogical that 48 mg/kg of SB-277011-B had no effect on the activation of mTOR or Akt in the BLA after the reinstatement of cocaine CPP when the lower dose tested in this investigation altered them. Nonetheless, other psychoactive drugs such as tricyclic antidepressants are known to exert their effects in a restricted therapeutical window and to have no efficacy when administered at lower or higher doses [64]. A possible explanation to these results may be that the ability of D3R to prevent the reactivation of cocaine-CPP triggered by psychological stress would result from a combined modulation of Akt/mTOR and MEK/ERK_1/2_ pathways achieved with the higher dose of its antagonist, and that this equilibrium would differ in both the hippocampal and amygdalar areas implicated in memory retrieval analyzed in this work. In agreement with discrepancies in the effects of D3R antagonists in distinct brain areas, it has been recently reported that the processing of aversive emotional stimuli is subject to different modulation by D3R in distinct brain nuclei of abstinent, drug-dependent subjects [65]. While in the BLA, the decrease in the activation of Akt/mTOR pathway appears to be compensated by un upregulation of ERK_1/2_, present data do not allow us to infer a clear modulation of Akt/mTOR and MEK/ERK_1/2_ pathways in the DG. Additionally, a role of the AC-cAMP-PKA or other signaling pathways in these processes, that are also modulated by D3R [51], cannot be excluded. Hence, further investigation is necessary to better understand these processes, such as broader dose–response studies of SB-277011-A effects on the stress-induced reactivation of cocaine-CPP and the underlying activity of AC-cAMP-PKA, Akt/mTOR, and/or MEK/ERK_1/2_ pathways, among others, in these nuclei.

Our data do not support the activities of any of the kinases analyzed in the BLA nor the DG as indicators of relapse in drug-seeking behavior. However, the blood levels of corticosterone after the blockade of D3R, to avoid the relapse in cocaine-CPP induced by psychological stress, might be relevantly related with the phosphorylation ratio of mTOR and Akt in the BLA and DG. Despite the prevention of the augmented corticosterone blood concentration induced by both psychological and physiological stress with 24 and 48 mg/kg of SB-277011-B, only the higher dose of the D3R blocker was able to antagonize the reinstatement of this behavior. These data might indicate that the dopaminergic neurons in the mesolimbic system would be strongly activated and consequently, would release high levels of DA in their projection areas. Therefore, a high dose of the D3R blocker would be needed for its antagonism and thus, the prevention of stress-induced reactivation of cocaine-CPP.

It has been reported that SB-27011-A administration does not alter spontaneous or stimulant-induced locomotion in basal conditions [66]. Furthermore, other D3R-selective-antagonist administrations to mice at doses that inhibited drug-seeking behaviors and drug-induced hyperlocomotion have been reported to not modify locomotor activity of mice in the absence of the abused substance [25,67,68,69]. Similarly, we observed lower locomotor activity during the reinstatement test of tail-pinch-induced cocaine-CPP after the administration of SB-277011-A. SB-277011-A has an 80- to 100-fold higher selectivity than other DA receptors and possesses strong affinity for human (pKi 7.95) and rat (pKi 7.97) D3R [70]. Concordantly, Collins et al. demonstrated SB-277011-A selectivity up to 56 mg/kg not only over D2R, but also over certain serotonergic and cholinergic receptors [71]. Thus, it is highly improbable that the decreased activity observed after SB-277011-A administration is be due to the antagonism of D2 receptors. As stated above, as opposed to D2 antagonists, SB-277011-A (up to 90 mg/kg, PO) and other selective D3R blockers failed to affect spontaneous locomotion [25,67,68,69] and did not provoke catalepsy [66] or sedation [72]. In agreement with this, Reavill et al. [73] reported that this antagonist was non-cataleptogenic and did not raise plasma prolactin levels at doses up to 78 mg/kg of the antagonist. On the other hand, Ross et al. [74] showed that the reduced distance travelled by animals in a nicotine self-administration model after the administration of 56 mg/kg of SB-277011-A was not observed when lower doses of this antagonist were administered. As our dosage only went up to 48 mg/kg, it is very unlikely that the effects observed in our study would be exerted through indirect off-target mechanisms. In addition, in accordance with the involvement of D3R in motivated behavior, as-yet-unpublished data of our laboratory point out that the lower locomotion during drug-induced behavior provoked by D3R blockade is likely due to decreased motivation. On the other hand, we found correlations between the locomotor activity of tail-pinched mice with the activities of ERK_1/2_ in the BLA and mTOR in the DG when the higher dose of the antagonist was administered. Yet, the mechanisms underlying what appears to be an attenuating effect of SB-277011-A on locomotion during the psychological- and physiological stress-induced reinstatement of cocaine-CPP cannot be easily interpreted on the basis of the present data.

### Limitations of the Current Study

Despite the broad literature supporting the high selectivity and affinity of SB-277011-A for D3R [70,71] and the lack of D2R-mediated effects of this and other D3R antagonists [25,66,67,68,69,72,73,74], in order to more clearly dismiss the locomotor effects of SB-277011-A, it should be tested at both 24 and 48 mg/kg doses.

## 4. Materials and Methods

### 4.1. Animals

All animals (male C57BL/6 mice) were provided by Charles River laboratories (Saint-Germain-sur-l’Arbresle, France), were at 6 weeks of age when they arrived at the laboratory, and were stabled in groups of four, in polypropylene cages (25 L × 25 W × 14.5 H cm) in a room with controlled temperature (22 ± 2 °C) and humidity (50 ± 10%), with ad libitum access to food and water (*n* = 73). Animals were adapted to a reversed 12 h light–dark cycle (lights off: 08:00 h–20:00 h) for 7 days before the beginning of the experiments. Mice were handled for a week before the beginning of the experiment. All surgical and experimental procedures were performed in accordance with the European Communities Council Directive of 22 September 2010 (2919/63/UE) and were approved by the local committee for animal research (Comité de Ética y Experimentación Animal; CEEA; RD 53/2013). Protocols were designed according to 3R to reduce the maximum number of mice and to minimize their suffering.

### 4.2. Drugs

Cocaine HCl (Alcaliber, Madrid, Spain) was dissolved in sterile 0.9% NaCl. The antagonist D3R SB-277011-A (N-[trans-4-[2-(6-Cyano-3,4-dihydro-2(1H)-isoquinolinyl)ethyl]cyclohexyl]-4-quinolinecarboxamide dihydrochloride; Tocris, St. Louis, MO, USA) was dissolved in deionized distilled water (vehicle). All drugs were administered intraperitoneally (i.p.) at a concentration of 0.01 mL/g body weight.

### 4.3. Conditioned Place Preference Paradigm

CPP paradigm was used to measure the rewarding properties of cocaine, as described previously [34]. The equipment used (Panlab, Barcelona, Spain) consisted of a box with two equally sized chambers (20 L × 18 W × 25 H cm) connected by a corridor (20 L × 7 W × 25 H cm). The two larger chambers differed in their wall paint and floor texture and provided distinct contexts (visual and tactile cues). The walls of the corridor were transparent to reduce the time spent in this compartment. Manual guillotine doors were inserted during the conditioning sessions and removed during the tests. Weight transducer technology and PPCWIN software were used to detect and analyze animal position and number of entries in each compartment during the test.

The CPP paradigm consisted of three different phases: a preconditioning phase, a conditioning phase, and a testing phase. Firstly, during the pre-conditioning phase, animals freely explored all compartments for 15 min and the time spent in each compartment was analyzed. Those showing a natural preference (>67%) or aversion (<33%) for any of the compartments were excluded from the experiment. All the behavioral experiments were carried out at the same time of day.

Mice were counterbalanced for compartment assignment in terms of initial spontaneous preference. A Student’s *t*-test was used to confirm that there were no significant differences in terms of time spent in compartments between groups (in cocaine-paired and saline-paired compartments) during pre-conditioning. The following day, during the conditioning phase, doors were closed to prevent animals from moving to other chambers.

All animals were conditioned with 25 mg/kg of cocaine on days 1 and 3 during 30 min. After that, they went back to the home cage and 4 h later, they received a saline injection before being conditioned to the vehicle-paired compartment for another 30 min. A dose of 25 mg/kg of cocaine has been previously shown to be adequate to induce a strong CPP [10,27,34,75]. On days 2 and 4, mice received first, a saline injection before being confined to the vehicle-paired chamber and 4 h after returning them to the home cage, animals were injected with cocaine before confinement in the drug-paired chamber for another 30 min. After that, on day 5, a post-conditioning test was carried out exactly as the pre-conditioning test: animals freely exploring all compartments for 15 min. On day 6 and for the following 7–8 weeks animals underwent extinction sessions twice a week on no consecutive days. During these sessions mice freely explored all the compartments for 15 min, as in pre-conditioning and post-conditioning tests. The criterion for the extinction of drug seeking behavior was considered when there were no significant differences (Student’s *t*-test) in the time spent by each group in the cocaine-associated chamber during the extinction test in comparison with that in the pre-conditioning test. To validate the extinction, a new session was repeated 48 h later.

To study the effect of acute psychological or physiological stress on the reinstatement of cocaine-induced CPP, 2 days after extinction was confirmed, animals were subjected to restraint or tail pinching, respectively, followed by a reinstatement test to analyze the relapse in cocaine-induced CPP. In this reinstatement test, mice were placed in the central corridor of the CPP apparatus during 15 min and were allowed to freely explore all the compartments (as in pre-conditioning, post-conditioning, and extinction tests).

All the stressful episodes were carried out in a different room to where the CPP apparatus was placed. To induce restraint stress, mice were placed in cylindrical plastic restrainers (2.5 cm diameter × 11.5 cm length) for 15 min, with an aperture in one of the ends to allow normal air flow. After that, mice were subjected to the reinstatement test. To induce the tail-pinch stress, animals were put in a plastic cage (25 H × 25 W × 14.5 cm) and a binder clip (7 mm wide, with oval inner section of 8 mm wide, and 13 mm height) was placed in the last third of the tip of the tail for 15 min (clamping force, 10 newton). After that, mice passed the reinstatement test in the CPP apparatus. For each stressor, a group of control animals were put in a plastic cage (25 H × 25 W × 14.5 cm) for 15 min without being subjected to any stressful stimulus for 15 min and then were tested for reinstatement of cocaine-CPP.

The role of D3R in the reactivation of cocaine-induced CPP was investigated by the administration of a single dose of the D3R antagonist, SB-277011-A (24 or 48 mg/kg) or its vehicle to mice 30 min before the stressful session.

Test data (time spent in each chamber of the apparatus and number of crossings between them) were recorded automatically with PPCWIN software (Panlab, Barcelona, Spain). As these data were collected by a computer, blinding to experimental group was not required.

### 4.4. Corticosterone Measurements

After behavioral experiments, mice were sacrificed via cervical dislocation, trunk blood samples were collected, and to separate the plasma, they were centrifuged at 960× *g* for 15 min at 4 °C. Samples were stored at −80 °C until their analysis. Corticosterone levels were determined using commercially available kits for mice (125 I-corticosterone radioimmunoassay; MP Biomedicals, Irvin, CA, USA). The sensitivity of the assay was 7.7 ng/mL.

### 4.5. Tissue Collection and Western Blot Analysis

Brains were removed and placed at −80 °C until they were processed. Brain interest regions were cut in 500 µm coronal slides on a cryostat at −20 °C and stored at −80 °C. BLA and DG were cut in two consecutive slides corresponding approximately −1.47 to 2.19 mm from Bregma for DG and −0.95 to −1.59 mm for BLA, according to the atlas of Franklin and Paxinos (2007) [76]. Bilateral 1 mm2 punches of the DG and BLA were stored into tubes containing 50 µL of homogenization solution (phosphate-buffered saline (PBS), sodium lauryl sulfate, proteases inhibitors, and a phosphatase-inhibitor cocktail set), as previously described by Leng et al. (2004) [77]. Tubes containing punches were consequently frozen in dry ice and place at −80 °C until processing. Samples were sonicated, vortexed, and sonicated again before centrifugation at 11,000× *g* for 10 min at 4 °C; then, the supernatant was recovered and placed at −80 °C. BCA method was used for protein concentration determination. An amount of 20 µg of protein samples were loaded and separated on 4–12% Criterion Bis-Tris Gels (EMD Millipore Corporation, Burlington, MA, USA) electrophoresis and transferred to polyvinylidene fluoride (PVDF) membranes (EMD Millipore Corporation). Membranes were saturated with 1% BSA in TBST (tris buffer saline tween, 0.15%) and, respectively, incubated overnight with primary and their corresponding secondary antibodies [rabbit anti p-mTOR (1:1000; #5536, Cell Signaling Technology, Danvers, MA, USA); rabbit anti mTOR (1:1000; #2983, Cell Signaling Technology), rabbit anti p-Akt (1:750; #4060L, Cell Signaling Technology), rabbit anti Akt (1:1000; #9272, Cell Signaling Technology), mouse anti p-ERK_1/2_ (1:750; #sc-7383, Santa Cruz Biotechnology, Santa Cruz, CA, USA), rabbit anti ERK2 (1:1000; sc-154, Santa Cruz), anti-rabbit antibody (1:10,000; #31430; Thermo Fisher Scientific, Waltham, MA, USA), anti-mouse antibody (1:10,000; #31460; Invitrogen, Waltham, MA, USA)]. Immunoreactivity was detected with an enhance chemiluminescent Western Blot detection system (ECL Plus, Thermo Fisher Scientific) and visualized using an Amersham Imager 680 (General Electric, Boston, MA, USA). Antibodies were stripped from the blots via incubation with stripping buffer (glycine, 25 mM and SDS 1%, pH 2) for 30 min at 37 °C. Results were normalized to β-actin, and quantifications were performed using ImageQuant TL 8.1 software (General Electric). The ratios of p-Akt/β-actin, Akt/β-actin, p-mTOR/β-actin, mTOR/β-actin, p-ERK_1/2_/β-actin and p-Akt/Akt, p-mTOR/mTOR, p-ERK_1/2_/ERK were plotted and analyzed. Protein levels were corrected for individual levels.

### 4.6. Statistical Analysis

All descriptive data were presented as means and standard error of means (S.E.M). To consider the extinction in behavioral experiments, a paired Student’s *t*-test was performed to analyze the difference between the time spent by mice in the cocaine-paired compartment during the pre-conditioning and post-conditioning tests. For the time spent in the cocaine-paired chamber, the statistical analysis was performed using one-way ANOVA with repeated measures followed by Tukey’s multiple comparisons test to determine specific group differences. Number of entries during extinction and reinstatement tests were analyzed through a paired Student’s *t* test. The reinstatement score, plasma corticosterone levels and Western blot data were analyzed using one-way ANOVA followed by the Tukey post hoc test. Effect size of corticosterone measurements was calculated through Cohen’s *d* coefficient. Correlations between different parameters were assessed using the Pearson’s correlation coefficient. Differences with a P < 0.05 were considered significant. Statistical analyses were performed with GraphPad Prism 9 (GraphPad Software Inc., San Diego, CA, USA).

## 5. Conclusions

In conclusion, the strength of this study is the hypothesis of a critical function of D3R in the reactivation of drug-seeking behaviors through distinct regulation of Akt/mTOR and MEK/ERK_1/2_ pathways in brain nuclei implicated in emotional and contextual memory consolidation and recall, thus supporting the promising therapeutic potential of its antagonists in preventing the stress-induced relapses. Our work might contribute to determining how D3R modulates the activity of brain areas implicated in the retrieval of cocaine-associated rewarding memories underpinning relapses. Nevertheless, this investigation leaves unresolved questions such as the mechanism by which D3R regulates glucocorticoid release or locomotor activity during a relapse in cocaine use, which remains to be elucidated by future investigations.

## Data Availability

The data are available from the corresponding authors on reasonable request.
